# Comparison of Destructive Strength Testing with Non-Destructive Ultrasonic Pulse Velocity Testing for Waste Marble Aggregate Concrete: An Experimental and Statistical Investigation

**DOI:** 10.3390/ma19143130

**Published:** 2026-07-21

**Authors:** Esra Tuğrul Tunç

**Affiliations:** Civil Engineering Department, Engineering Faculty, Firat University, 23119 Elazig, Turkey; esratugrul@firat.edu.tr

**Keywords:** waste marble aggregate concrete, concrete strength, ultrasonic pulse velocity test, destructive testing, non-destructive testing, statistical analysis

## Abstract

In this study, the performance of eco-friendly concrete produced by utilizing waste marble as a total aggregate replacement was evaluated. The experimental findings indicated that the developed waste marble aggregate concrete (WMC) specimens successfully met the standardized strength requirements for structural applications. The main objective of this investigation was to determine the experimental and statistical correlation between destructive strength tests and the non-destructive ultrasonic pulse velocity (UPV) test, taking into account the content ratios of concrete. This study presents an experimental and statistical investigation to correlate destructive strength properties with non-destructive UPV measurements in eco-friendly concrete. A total of 300 concrete cubic specimens were produced by fully substituting conventional aggregates with waste marble aggregates across five distinct water-to-cement ratios (*W*/*C* = 0.20 to 0.40) and ten aggregate-to-cement ratios (*WMA*/*C* = 1.1 to 2.0). Compressive strength (*f_c_*), splitting tensile strength (*f_t_*), and UPV tests were conducted on the 28th day. The experimental results showed that *f_c_* ranged from 19.2 to 37.5 MPa, *f_t_* from 2.3 to 4.4 MPa, and UPV from 3580 to 4386 m/s, confirming the high structural quality of the waste marble aggregate concrete. Non-linear regression analyses were performed using IBM SPSS Statistics 22 to develop empirical models predicting destructive strengths based on mix design parameters and UPV data. The proposed statistical models demonstrated high accuracy with determination coefficients (R^2^) of 0.98 for *f_c_* and 0.97 for *f_t_*, backed by low mean absolute relative deviations (6% and 8%, respectively). The findings indicate that the developed empirical formulations can reliably evaluate the strength of WMC in a practical and non-destructive manner.

## 1. Introduction

Waste management is an ever-evolving research topic in the world. Today, due to the increasing population, the amount of waste is increasing and poses a great threat to the environment. The aim of recycling, which is necessary to reduce the damage caused by waste, is to reduce the amount of waste that has become an environmental problem and to gain economic benefits by using this waste. Recent scientific studies have focused on the recycling of waste materials [1,2,3]. In this context, the utilization of marble, whose abundant waste is left in the environment, both in coarse and fine powder form in concrete production, attracts the interest of application engineers. Turkey, where the current study was conducted, has 13.9 billion tons of total marble reserves and is capable of meeting approximately 80 years of world marble consumption [4,5]. In this respect, the utilization of marble wastes in the production of concrete with the desired quality in Turkey, as in the whole world, will be of great benefit in terms of the environment and economy.

Low compressive strength significantly affects the behavior of concrete, or in other words, reinforced concrete. As is known, concrete is a brittle material; the energy absorption (toughness) and tensile strength of concrete are quite low [6,7,8]. In a reinforced concrete system, concrete shows a more ductile behavior as a result of different reinforcements placed in the concrete. However, if the compressive strength of concrete is low, this situation causes the adherence between concrete and reinforcement to be insufficient even at low stresses. In this case, longitudinal reinforcements easily peel off from the concrete, and the longitudinal reinforcements connected with dense stirrups buckle. Subsequently, the concrete breaks, the reinforcement breaks and the structure collapses. This situation poses a great danger, especially during an earthquake [9,10]. For this reason, concrete should be of the quality required by the relevant standards according to the place of use and should be monitored throughout the life of the structure. In this context, non-destructive tests for concrete strength are easier to apply than destructive tests, yield results quickly in the field, are more economical, can be measured even in large areas and do not damage concrete structures, making non-destructive test methods advantageous [11,12,13].

The most commonly used non-destructive methods for addressing concrete strength are the UPV Test, Windsor Probe Penetration Test Method, Schmidt Hammer Rebound Hardness Test, Radiographic Test Method, Ground Radar Method, and Ultrasonic Tomography Method [14,15]. It is of great importance to evaluate the quality of concrete with non-destructive methods, as the structural integrity and strength of concrete are critical for civil engineering. UPV testing is a reliable and effective method frequently used for this purpose [16,17]. However, in order for the test to produce accurate and reliable results, it should be performed in accordance with the relevant standards [18,19,20], and the results should be interpreted by experts. Comparison with the findings of compressive strength and splitting tensile strength of concrete and statistical analysis of the data are known to significantly increase the reliability of UPV test findings [21,22]. In the UPV test, high-frequency sound waves (ultrasonic waves) are passed through the concrete, and the time it takes for these waves to travel a certain distance is measured. From the time value obtained, the speed of the wave in the concrete is calculated. This velocity provides important information about the homogeneity, porosity and therefore the strength of the concrete [23,24,25].

In a hierarchical context, the adoption of UPV in concrete characterization has evolved from basic quality control assessments to sophisticated structural health monitoring and damage quantification. Mechanistically, the velocity of longitudinal ultrasonic waves directly correlates with the dynamic elastic modulus and bulk density of the cementitious matrix. Consequently, any alteration in the internal matrix—such as micro-cracking, localized void clusters, or changes in aggregate-paste interfacial zones—manifests as a distinct shift in wave attenuation and transit time. Recent progressive studies have successfully utilized UPV techniques to monitor the hydration kinetics of high-performance mixtures, evaluate frost damage resistance, and map early-age microstructural development. Furthermore, with the rise of sustainable development in civil engineering, researchers have increasingly applied non-destructive acoustic methods to quantify the internal compactness of concrete blending unconventional materials, such as recycled aggregates, slag, and industrial stone wastes. Because these eco-friendly materials often alter the matrix porosity and absorption characteristics, establishing progressive non-destructive baselines is essential to validate their structural reliability without relying solely on destructive core drilling. However, while the correlation between UPV and traditional concrete frameworks is well documented, a comprehensive synthesis combining multi-parametric mix designs (*W*/*C* and *WMA*/*C* ratios) with non-linear statistical modeling for total waste marble aggregate systems remains an open research frontier [19,21,25].

UPV is a non-destructive testing method that has a wide range of applications, such as flaw and defect detection, internal structure examination, reinforcement detection, modulus of elasticity estimation and thickness measurement. It is preferred over other non-destructive test methods because it can be used on very thick specimens and has a high accuracy ratio in detecting even very small defects. In addition, the fact that it can be measured from a single surface, fast measurement and fast results make the ultrasonic testing method more advantageous than other non-destructive testing methods. On the other hand, it is difficult to examine very small specimens with rough surfaces. In addition, defects or reinforcements that are too close to the concrete surface cannot be detected, and high technical knowledge, experience and deeper research are required to understand and interpret the test results [26,27,28].

Concrete has a heterogeneous structure consisting of different materials such as cement, water and aggregates [29]. Therefore, factors such as cement type, water-to-cement ratio, aggregate-to-cement ratio, aggregate type and size affect the internal structure of concrete and thus the UPV [30,31]. As ultrasonic waves travel through this structure, they interact with different components such as pores, cracks, and aggregates. Concretes with a denser and homogeneous structure have a higher ultrasonic wave velocity as they are less obstructive to the waves. This is usually associated with high compressive strength. Concretes with high compressive strength have a tighter structure, allowing ultrasonic waves to travel faster. This can be explained by the reduction of voids and heterogeneity in the internal structure of concrete [32,33].

In general, there is a positive correlation between the compressive strength of concrete and UPV. That is, as the compressive strength of concrete increases, the propagation velocity of ultrasonic waves in concrete also increases. This is because concretes with high strength values have a denser and more homogeneous structure. This allows ultrasonic waves to travel faster with fewer obstacles [34]. It has been stated that when non-destructive methods are used individually, they can be used safely when correlated with destructive tests to obtain more accurate results about the concrete compressive strength of existing structures [35]. Accordingly, in the present study, concrete compressive strength test results and concrete splitting tensile strength test results, which are destructive tests, were investigated by statistical correlation with UPV test values.

The acoustic behavior of WMC differs from ordinary concrete due to mineralogical and microstructural variances. Conventional concrete with natural aggregates exhibits predictable wave attenuation. However, waste marble aggregates consist primarily of crystalline calcite (CaCO_3_) with angular shapes, which increases internal wave scattering and alters transit times through the interfacial transition zone. Therefore, a specific UPV value in ordinary concrete does not reflect the same strength profile in WMC. The innovation of this study is the development of multi-parametric non-linear equations that integrate both UPV values and mix design proportions (*W*/*C* and *WMA*/*C*) to reliably capture the unique density and strength development of full-replacement WMC [4,5].

In recent years, structural engineering has shifted toward intelligent prediction frameworks, machine-learning-assisted non-destructive evaluations, and optimization-based assessment protocols. Comprehensive reviews underscore the growing integration of machine learning and deep learning models for automated structural health monitoring across civil engineering applications. Furthermore, advanced algorithmic variants, such as improved gradient boosting models, have demonstrated high efficiency in capturing localized structural behaviors, including strain distributions in advanced fiber-reinforced polymer strengthening systems. While this study prioritizes direct mathematical transparency via non-linear empirical regression for field operators, these emerging AI-driven methodologies represent the next evolutionary step for large-scale, automated quality control frameworks in sustainable infrastructure [36,37].

The acoustic behavior of WMC differs from ordinary concrete due to mineralogical and microstructural variances. Conventional concrete with natural aggregates exhibits predictable wave attenuation through a standard siliceous or basaltic matrix. In contrast, waste marble aggregates consist primarily of dense crystalline calcite (CaCO_3_) with highly angular shapes and distinct cleavage planes. These geometric boundaries increase internal wave scattering and alter transit times across the localized interfacial transition zone under varying moisture levels (4,5). Consequently, a specific UPV value in ordinary concrete does not reflect the exact same mechanical strength profile in WMC. The innovation of this study is the development of multi-parametric non-linear equations that integrate both UPV values and concrete mix design proportions to reliably capture the unique internal density and velocity shifting of full-replacement WMC systems.

The practical novelty of this study addresses a specific gap in the non-destructive evaluation of full-replacement WMC. Conventional empirical models typically rely on single-variable formulations. These simple correlations often fail because they neglect internal wave scattering and path tortuosity caused by the angular geometry and crystalline calcite microstructure of marble grains under fluctuating binder volumes. This study advances existing modeling methods by introducing a multi-parametric, non-linear regression matrix that links acoustic propagation directly to physical mix proportions. This mathematical integration provides high-accuracy formulations that serve as an immediate quality control tool for field engineers, allowing accurate strength estimation without requiring destructive core extraction.

Within the scope of the present study, a series of WMC specimens were produced for UPV testing, which is one of the reliable non-destructive testing methods used in concrete strength determination and structural defect detection. The concrete specimens produced for different water-to-cement ratios and aggregate-to-cement ratios were subjected to compressive strength, splitting tensile strength and UPV tests. The primary objective of this study was to establish robust empirical and statistical correlations between destructive strength indicators (*f_c_* and *f_t_*) and non-destructive UPV measurements for sustainable concrete formulated entirely with waste marble aggregates. To achieve this, a systematic experimental program was conducted on 300 cubic specimens, systematically varying the water-to-cement (*W*/*C*) and aggregate-to-cement (*WMA*/*C*) ratios to capture a broad range of structural performances. The gathered experimental dataset was then subjected to multi-parametric non-linear regression analysis using IBM SPSS Statistics 22 (Version 22.0, IBM Corp., Armonk, NY, USA) to develop practical predictive formulations. Ultimately, this study demonstrates that incorporating predictive models that account for both acoustic wave velocity and mix design proportions yields highly accurate strength estimations (R^2^ ≥ 0.97), proving that non-destructive testing can reliably evaluate the structural integrity of full-replacement WMC frameworks.

## 2. Materials and Methods

### 2.1. Materials

**Figure 1:**
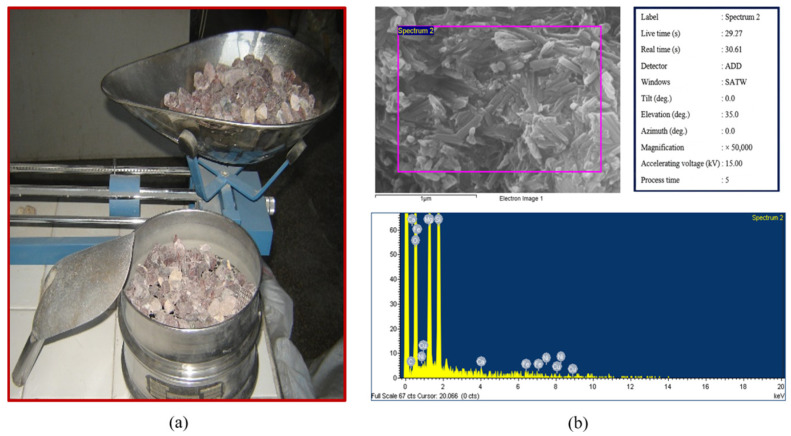
Tested waste marble aggregate: (**a**) view from the laboratory, (**b**) SEM picture and EDX analysis.

In this experimental study, waste marble aggregates with *D_max_* = 16 mm from the Elazig province of Turkey were used (Figure 1a). The waste marble aggregates were sieved, and the amount remaining on each sieve was stored separately and divided into the following groups: 0–1 mm, 1–2 mm, 2–4 mm, 4–8 mm, and 8–16 mm. The same adjusted granulometry was selected for the mixture aggregates to be used in the production of waste marble aggregate concrete, and the waste marble aggregate ratios to be included in the mixture were determined as 25% for 0–1 mm, 10% for 1–2 mm, 15% for 2–4 mm, 21% for 4–8 mm and 29% for 8–16 mm according to Fuller’s parabola [38,39]. In addition, the average saturated dry surface-specific gravity of waste marble mixture aggregates was measured as 2.53 g/cm^3^, water absorption ratio as 1.3%, and Los Angeles abrasion loss value as 32.5%.

With devices developed to detect the finest details in any material, it is now possible to examine the basic structure of electronic and optical systems by analyzing them at high magnifications. The scanning electron microscope (SEM) used for this purpose works on the principle of scanning the surface with high-energy electrons focused on a very small area. Thanks to SEM, micro- and nano-scale structures of solid materials can be determined. In addition, elemental analysis of the tested material structure can also be performed. Precise measurements can also be taken from non-smooth surfaces with SEM. However, since it provides three-dimensional imaging and elemental analysis, it is a highly preferred device in this direction. Therefore, it is clear that the reliability of the analysis performed with SEM will be high [40,41].

Scanning electron microscopy (SEM) was performed using an energy-dispersive X-ray (EDX) micro analyzer (Oxford Instruments, Abingdon, UK) to examine the microstructure of the waste marble aggregate. The magnification of the SEM image was chosen to be ×50,000 for more detailed examination. When the basic structure of the tested waste marble aggregate was examined, low-porosity microstructure and microcracks with relatively small thickness despite the ×50,000 magnification were observed. In particular, the high concentration of Fe, Ca, Mg, and Si elements indicates a crystalline mineralogical framework that supports a stable matrix structure. This assessment is consistent with previous literature investigations [42,43]. Consequently, these microstructural properties suggest that the waste marble aggregate provides a highly compact structure suitable for concrete production.

The SEM micrographs and EDX spectra illustrated in Figure 1 reveal the foundational microstructure of the waste marble aggregate (*WMA*). The matrix is dominated by highly rigid, crystalline calcite (CaCO_3_) blocks with angular geometric boundaries. This crystalline structure plays a dual role in governing the macroscopic properties of the final concrete. Microstructurally, these angular calcitic grains function as highly effective non-reactive fillers that physically densify the interfacial transition zone and reduce interconnected capillary porosity. This internal compaction directly optimizes acoustic wave propagation; in contrast to conventional aggregates that scatter stress waves across micro-voids, the uniform and dense *WMA* matrix allows ultrasonic pulses to pass through a direct solid pathway, resulting in high baseline UPV values (>3500 m/s). Furthermore, under destructive mechanical loading, this tight crystalline bonding along the interfacial transition zone forces microcracks to follow highly tortuous propagation paths around or directly through the rigid calcitic grains, preventing premature matrix failure and directly supporting the stable compressive and splitting tensile strength trends observed in the developed mix designs.

It is known that the most important factor that increases the strength of concrete is the use of cement in large amounts and with high strength [44,45]. Therefore, it is very important to select the appropriate cement for the purpose of producing concrete with the desired properties. Within the scope of the present study, the type of cement used in the production of the experimentally tested concrete specimens is CEM I 42.5 R Portland cement (Elazığ Cement Factory, Elazığ, Turkey).

The amount of water required for the hydration of the cement was determined as the minimum amount of water required to achieve the required workability. This amount of water was determined to be 25% of the cement weight for conventional concrete [46,47,48]. In the present experimental study, mix water that was checked for compliance with the relevant standard [49] was used.

### 2.2. Experimental Methods

In the present study, a series of WMC specimens was produced for experimental tests considering different water-to-cement ratios (*W*/*C*) and different waste-marble-aggregate-to-cement ratios (*WMA*/*C*). Fifty series were evaluated; WMC1-WMC10 for *W*/*C* = 0.20, WMC11-WMC20 for *W*/*C* = 0.25, WMC21-WMC30 for *W*/*C* = 0.30, WMC31-WMC40 for *W*/*C* = 0.35 and WMC41-WMC50 for *W*/*C* = 0.40. Three control specimens were produced in each series, and the values considered were the averages of the strength values of these three control specimens. A total of 300 150 × 150 × 150 mm concrete cubic specimens were produced, with 150 tested for concrete compressive strength (*f_c_*) and 150 tested for concrete splitting tensile strength (*f_t_*). A UPV test was applied to these specimens before the strength tests were performed, and the average of the measured values was recorded as the UPV for each specimen. To ensure data reliability, the within-test coefficient of variation was calculated across all batches. The coefficient of variation thresholds remained under 5% for the mechanical strength indicators and under 1.5% for the acoustic measurements, confirming low experimental uncertainty and high specimen homogeneity. In this context, a detailed flow chart for both experimental and statistical studies is presented in the graphical abstract.

In the present study, five different water-to-cement ratios (*W*/*C* = 0.20, 0.25, 0.30, 0.35 and 0.40) and ten different waste-marble-aggregate-to-cement ratios (*WMA*/*C* = 1.1, 1.2, 1.3, 1.4, 1.5, 1.6, 1.7, 1.8, 1.9 and 2.0) were tested to produce a series of WMC specimens for different mix ratios. These wide-ranging proportions were selected to systematically capture the full acoustic and mechanical performance boundaries of the calcitic matrix without chemical admixtures. For the production of each series of concrete specimens, waste marble aggregate, cement and water were weighed and prepared in advance. The specimens were subjected to concrete compressive strength, concrete splitting tensile strength and UPV tests. For the production of each series of concrete specimens, waste marble aggregate, cement and water were weighed and prepared in advance. In order to objectively determine the effect of waste marble aggregate on the destructive and non-destructive strength of concrete, no mineral/chemical admixtures were used. A laboratory-type mixer with a 125 dm^3^ capacity and a horizontal axis was used for mixing the fresh concrete.

The experimental program utilized a total of 300 concrete specimens divided into two separate series of 150 specimens. The first series of 150 specimens was tested sequentially, undergoing non-destructive UPV measurements immediately before the destructive compressive strength (*f_c_*) tests. The second series of 150 specimens was designated solely for splitting tensile strength (*f_t_*) testing to evaluate the tensile performance of the WMC matrix independently.

There is no definite rule by the relevant standards on how to place the materials that will form the content of WMC specimens into the mixer by determining the mixing ratios. However, according to the experiences obtained from the applications, the order of placing the materials in the mixer and the method applied affect the homogeneity of the concrete to be obtained to a certain extent [50,51]. In this direction, the method deemed appropriate for the preliminary trial experiments carried out before the study is as follows. First, coarse and fine aggregates were put into the mixer and mixed for 2 min; then, two-thirds of the mixing water was added while the mixer was running, and the mixing process continued for another 3 min after the WMC specimen gained a homogeneous appearance. The fresh concrete was placed in 150 × 150 × 150 mm molds for related tests in accordance with the relevant standard [52]. The concrete was poured in two layers to ensure proper placement of the concrete in the molds. After each layer was poured, air bubbles were removed by vibration on the shaking table at certain intervals and the fresh concrete was placed in the molds properly.

**Figure 2:**
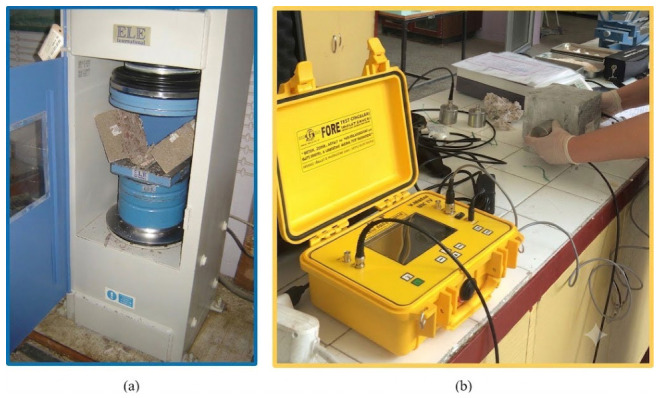
View of destructive and non-destructive experimental tests for WMC specimens: (**a**) strength tester, (**b**) UPV test device.

Following the casting process, all waste marble aggregate concrete (WMC) specimens were kept in their molds at room temperature (20 ± 2 °C) for 24 h. After demolding, the specimens were immediately transferred to a standard water curing tank and completely submerged in lime-saturated water at a controlled temperature and a relative humidity of no less than 95%, in strict accordance with the relevant standard [53] EN 12390-2. The specimens remained under these uniform curing conditions for 28 days until the scheduled non-destructive and destructive testing dates. At the end of the 28th day, compressive strength tests were performed for the hardened 150 mm × 150 mm × 150 mm cubic WMC specimens in accordance with the relevant standard [53] in the 2500 kN capacity concrete test press device (ELE International, Leighton Buzzard, UK) (Figure 2a) in the existing laboratory. The specimens placed in the device were loaded at a constant velocity of 6.8 MPa/s, and after determining the fracture loads, the concrete compressive strength values (*f_c_*) were calculated by dividing the maximum load value (*P*) required for the specimen to fracture by the cross-sectional area (*A*) perpendicular to the direction of application of the load.

Similar to the concrete compressive strength test, the concrete splitting tensile strength test was carried out under the same conditions and in accordance with the relevant standard [54] for WMC cubic specimens of the same dimensions. The specimens placed in the device were loaded at a constant speed of 1.05 MPa/s, and the concrete splitting tensile strength tests of the specimens were calculated with the determined fracture loads. The concrete compressive strength values (*f_c_*) and concrete splitting tensile strength values (*f_t_*) obtained from the study are reported as the arithmetic mean of three control specimens for each series in Section 3 (Table 1).

At the end of the 28th day, the UPV value of 150 × 150 × 150 mm cubic WMC specimens was measured according to the relevant standard [18]. The UPV was measured with a Pundit-type ultrasonic measuring device (James Instruments Inc., Chicago, IL, USA/Fore Test, Ankara, Turkey) with a digital display in accordance with the relevant standard [55] at a frequency of 55 kHz (Figure 2b). Before starting the experimental test with the device, which was zeroed and calibrated before each test, the WMC specimens were placed on a flat and clean surface, and the surfaces were thoroughly cleaned with a soft brush. Ultrasonic gel was applied on the opposite surfaces where the transmitters and receivers of the device would be placed. The WMC specimens were sandwiched between the transmitter and receiver to eliminate air entrainment (Figure 2b). For each WMC specimen, the measurement was repeated twice by switching the probes, the UPV values of the WMC specimens were calculated with the help of Equation (1), and the average UPV values of 3 control specimens for each series were reported.

(1) $$V=\frac{L}{t}$$

In Equation (1), *V* = ultrasonic pulse velocity (m/s), *L* = specimen length (m) and *t* = ultrasound transit time (s).

### 2.3. Statistical Methods

In the statistical part of the present study, the experimental findings were evaluated, and statistical methods were developed to calculate the concrete compressive strength values (*f_c_*) and concrete splitting tensile strength values (*f_t_*) obtained by destructive strength tests depending on the mix proportions of WMC specimens and the UPV values obtained by non-destructive testing. For this purpose, IBM SPSS Statistics 22 (SPSS Inc., Chicago, IL, USA), an up-to-date and advanced analytical software, was utilized [56].

**Figure 3:**
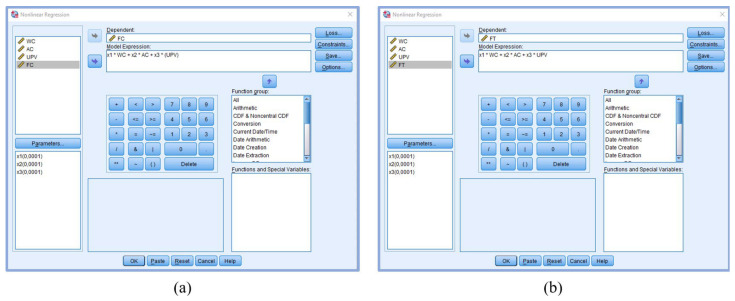
Images from the application of IBM SPSS Statistics 22 software for statistical methods developed with non-linear regression: (**a**) for statistical model output *f_c_*, (**b**) for statistical model output *f_t_.*

With IBM SPSS Statistics 22 software, statistical analyses such as data analysis, data management and data visualization can be easily performed, and statistical modeling/regression analyses based on various predictions can be performed successfully [57,58,59]. Using this software, which has many different computational capabilities, visualizations of the entry, processing and application methods of the statistical methods developed within the scope of the current study were created and are presented in Figure 3. In this context, in Figure 3a, non-linear regression analyses were performed for model inputs *W*/*C*, *WMA*/*C*, and UPV and for model output *f_c_*; in Figure 3b, non-linear regression analyses were performed for model output *f_t_* with the same model inputs, and analysis of variance (ANOVA) was performed.

## 3. Experimental Study Results and Discussion

In the experimental part of the present study, the results of the concrete compressive strength values (*f_c_*), concrete splitting tensile strength values (*f_t_*), and UPV values for the WMC specimens produced with different *W*/*C* and different *WMA*/*C* dimensionless parameters are presented in Table 1. Table 1 shows that the *f_c_* values of the 50 series WMC specimens ranged from 19.2 to 37.5 MPa, the *f_t_* values ranged from 2.3 to 4.4 MPa, and UPV values ranged from 3580 to 4386 m/s.

**Figure 4:**
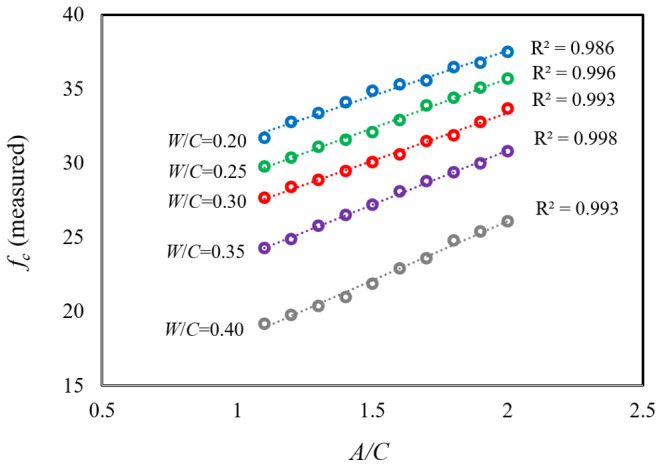
Variation in the *f_c_* values with *WMA*/*C* ratios for fixed *W*/*C* ratios.

According to Figure 4, when the variation in the concrete compressive strength values (*f_c_*) for fixed *W*/*C* ratios with different *WMA*/*C* ratios of WMC specimens is examined, it is observed that as the *WMA*/*C* ratios increase, the *f_c_* values increase in a nearly linear manner. In this context, the correlation coefficient R^2^ > 0.99 for the fitted linear curves is observed, and it can be said that the relationship is in very good agreement. For waste-marble-aggregate-to-cement ratios ranging between *WMA/C* = 1.1–2.0, *f_c_* = 31.7–37.5 MPa for *W*/*C* = 0.20, *f_c_* = 29.8–35.7 MPa for *W*/*C* = 0.25, *f_c_* = 27.7–33.7 MPa for *W*/*C* = 0.30, *f_c_* = 24.3–30.8 MPa for *W*/*C* = 0.35 and *f_c_* = 19.2–26.1 MPa for *W*/*C* = 0.40. For fixed *W*/*C* ratios, it is observed that for an approximately 82% increase in *WMA*/*C* ratios, there is an increase in *f_c_* values by 18% for *W*/*C* = 0.20, 20% for *W*/*C* = 0.25, 22% for *W*/*C* = 0.30, 27% for *W*/*C* = 0.35 and 36% for *W*/*C* = 0.40. This shows that for higher *W*/*C* ratios, *f_c_* values increase more as the *WMA*/*C* ratio increases.

When the change in *f_c_* values with *W*/*C* ratios was analyzed, it was determined that *f_c_* values decreased by approximately 6% on average when *W*/*C* increased from 0.20 to 0.25, *f_c_* values decreased by approximately 7% on average when *W*/*C* increased from 0.25 to 0.30, *f_c_* values decreased by approximately 9% on average when *W*/*C* increased from 0.30 to 0.35 and *f_c_* values decreased by approximately 18% when *W*/*C* increased from 0.35 to 0.40. As the *W*/*C* ratio increased from 0.20 to 0.40, it was calculated that the *f_c_* values decreased by approximately 35% on average. However, it was calculated that the *f_c_* values increased by approximately 25% on average while the *WMA*/*C* ratio increased from 1.1 to 2.0. Thus, it is clearly understood that the water-to-cement ratio (*W*/*C*) and aggregate-to-cement ratio (*WMA*/*C*) have a great effect on concrete strength. Thus, it was determined that *f_c_* values decreased as the *W*/*C* ratio increased and *f_c_* values increased as the *WMA*/*C* ratio increased.

**Figure 5:**
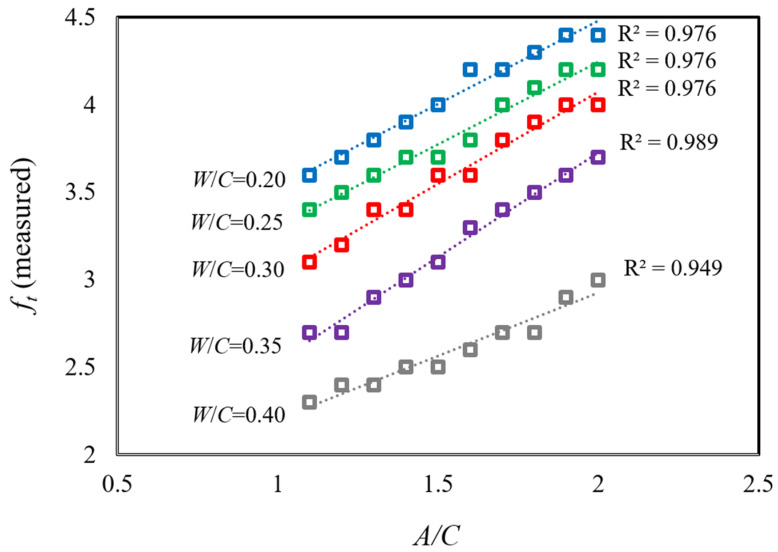
Variation in *f_t_* values with *WMA*/*C* ratios for fixed *W*/*C* ratios.

When Figure 5 is examined, it is observed that as the *WMA*/*C* ratios increase for fixed *W*/*C* ratios of WMC specimens, the concrete splitting tensile strength values (*f_t_*) also increase nearly linearly, and the correlation coefficient R^2^ > 0.98 for the fitted linear curves. Thus, as in the case of *f_c_* values, it is possible to say that the relationship between *WMA*/*C* values and *f_t_* values has a very good fit. For waste-marble-aggregate-to-cement ratios ranging between *WMA*/*C* = 1.1–2.0, *f_t_* = 3.6–4.4 MPa for *W*/*C* = 0.20, *f_t_* = 3.4–4.2 MPa for *W*/*C* = 0.25, *f_t_* = 3.1–4.0 MPa for *W*/*C* = 0.30, *f_t_* = 2.7–3.7 MPa for *W*/*C* = 0.35 and *f_t_* = 2.3–3.0 MPa for *W*/*C* = 0.40. As can be seen, for fixed *W*/*C* ratios, it is observed that for an approximately 82% increase in *WMA*/*C* ratios, there is an average increase in *f_t_* values of 22% for *W*/*C* = 0.20, 24% for *W*/*C* = 0.25, 29% for *W*/*C* = 0.30, 37% for *W*/*C* = 0.35 and 30% for *W*/*C* = 0.40.

When the change between *W*/*C* and *f_t_* values was analyzed, it was determined that *f_t_* values decreased by approximately 6% on average while *W*/*C* increased from 0.20 to 0.25, *f_t_* values decreased by approximately 6% on average while *W*/*C* increased from 0.25 to 0.30, *f_t_* values decreased by approximately 11% on average while *W*/*C* increased from 0.30 to 0.35 and *f_t_* values decreased by approximately 18% while *W*/*C* increased from 0.35 to 0.40. It was calculated that *f_t_* values decreased by approximately 36% on average as the *W*/*C* ratio increased from 0.20 to 0.40. This ratio is very close to the ratio of change in the *f_c_* value with *W*/*C*. In addition, it was calculated that ft values increased by approximately 28% on average while the *WMA*/*C* ratio increased from 1.1 to 2.0. Thus, it was determined that the change in *f_t_* values with *W*/*C* and *WMA*/*C* ratios was quite similar to the change in *f_c_* values with the said ratios. Thus, it was concluded that, as with *f_c_* values, *f_t_* values decreased as the *W*/*C* ratio increased, while *f_t_* values increased as the *WMA*/*C* ratio increased.

UPV value is an important parameter obtained by non-destructive measurement that provides an idea of the quality and strength of the tested concrete. A UPV value of less than 2000 m/s indicates a very poor-quality concrete, between 2000 and 3000 m/s indicates a poor-quality concrete, between 3000 and 3500 m/s indicates a concrete of doubtful quality, between 3500 and 4500 m/s indicates a quality concrete, and greater than 4500 m/s indicates a very high-quality concrete [60]. In this respect, when the UPV values measured in the present study are analyzed, it can be said that they vary between 3580 and 4386 m/s, and all WMC specimens produced have sufficient quality and strength according to the place of use.

**Figure 6:**
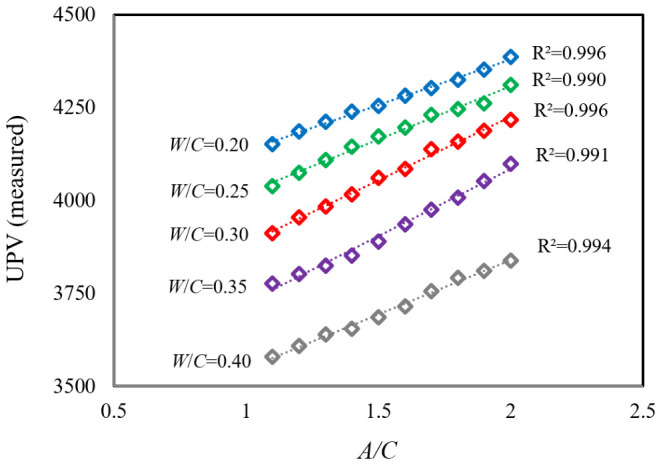
Variation in UPV values with *WMA*/*C* ratios for fixed *W*/*C* ratios.

In Figure 6, when the variation in UPV values of WMC specimens with *WMA*/*C* ratios for fixed *W*/*C* ratios is examined, it is observed that as the *WMA*/*C* ratios increase, UPV values increase in a nearly linear manner. The correlation coefficient of the linear fit for this increasing trend is R^2^ > 0.99, indicating that the relationship is a very good fit. For waste-marble-aggregate-to-cement ratios ranging between *WMA*/*C* = 1.1–2.0, UPV = 4152–4386 m/s for *W*/*C* = 0.20, UPV = 4038–4311 m/s for *W*/*C* = 0.25, UPV = 3192–4217 m/s for *W*/*C* = 0.30, UPV = 3776–4098 m/s for *W*/*C* = 0.35 and UPV = 3580–3838 m/s for *W*/*C* = 0.40. As can be seen, for fixed *W*/*C* ratios, it is observed that for an approximately 82% increase in *WMA*/*C* ratios, UPV values increase approximately 6% for *W*/*C* = 0.20, 7% for *W*/*C* = 0.25, 8% for *W*/*C* = 0.30, 9% for *W*/*C* = 0.35 and 7% for *W*/*C* = 0.40. Thus, it is seen that the ratio of increase in UPV values is less than the ratio of increase in strength values.

When the change in UPV values with *W*/*C* was analyzed; it was determined that UPV values decreased approximately 2% on average when *W*/*C* increased from 0.20 to 0.25, UPV values decreased approximately 3% on average when *W*/*C* increased from 0.25 to 0.30, UPV values decreased approximately 4% on average when *W*/*C* increased from 0.30 to 0.35 and UPV values decreased approximately 5% when *W*/*C* increased from 0.35 to 0.40. It was calculated that the UPV values decreased by approximately 13% on average as the *W*/*C* ratio increased from 0.20 to 0.40. However, it was calculated that the UPV values increased by approximately 7% on average while the *WMA*/*C* ratio increased from 1.1 to 2.0. Thus, similar to the strength values, it was concluded that UPV values decreased as the *W*/*C* ratio increased and that UPV values increased as the *WMA*/*C* ratio increased, but the ratios of change were relatively low.

**Figure 7:**
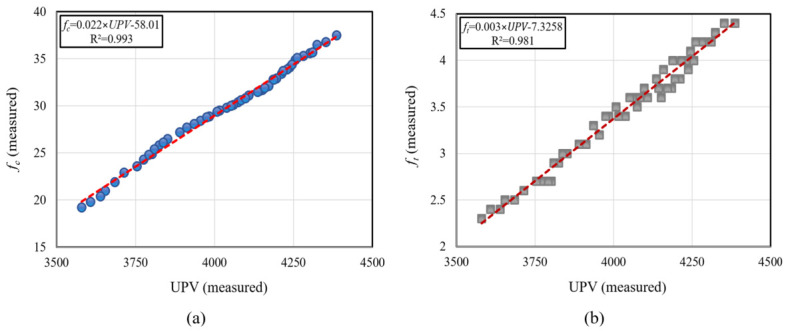
Variation in experimentally measured concrete strength values with UPV values: (**a**) for *f_c_*, (**b**) for *f_t_*. (The markers indicate measured experimental data points, and the red dashed lines represent the fitted regression curves).

The variation in concrete compressive strength values (*f_c_*) with UPV values of the tested WMC specimens is presented in Figure 7a, and the variation in concrete splitting tensile strength values (*f_t_*) with UPV values of the tested WMC specimens is presented in Figure 7b. The experimental results show that the lowest UPV value (UPV = 3580 m/s), the lowest *f_c_* value (*f_c_* = 19.2 MPa) and the lowest *f_t_* value (*f_t_* = 2.3 MPa) were measured for the highest tested water-to-cement ratio (*W*/*C* = 0.40) and the lowest tested waste-marble-aggregate-to-cement ratio (*WMA*/*C* = 1.1). The highest UPV value (UPV = 4386 m/s), the highest *f_c_* value (*f_c_* = 37.5 MPa) and the highest *f_t_* value (*f_t_* = 4.4 MPa) were measured for the lowest tested water–cement ratio (*W*/*C* = 0.20) and the highest tested waste marble aggregate cement ratio (*WMA*/*C* = 2.0).

For the tested WMC specimens, UPV values change in the range UPV = 3580–4386 m/s, while concrete compressive strength values change in the range *f_c_* = 19.2–37.5 MPa. For an average increase of approximately 22.5% in UPV values, an average increase of approximately 95% in *f_c_* values was calculated. A near-perfect agreement between *UPV* and *f_c_*, with a coefficient of determination of 0.99 < R^2^ < 1.0, was observed, and Equation (2) was developed in this context (Figure 7a). For the tested WMC specimens, the variation in UPV values in the range of UPV = 3580–4386 m/s was observed in the range of *f_t_* = 2.3–4.4 MPa for the concrete splitting tensile strength values. For an average increase of approximately 22.5% in UPV values, an average increase of approximately 91% in ft values was calculated, close to the ratio of increase in *f_c_* values. A near-perfect agreement between UPV and *f_t_* values, with a coefficient of determination of 0.98 < R^2^ < 1.0, was observed, and Equation (3) was developed in this context (Figure 7b).

*f_c_* = 0.022 × *UPV*-58.01 (2)

*f_t_* = 0.003 × *UPV*-7.3258 (3)

While the current experimental framework establishes strong acoustic–mechanical correlations for full-replacement WMC, the practical engineering deployment of these mixtures requires careful consideration of long-term durability performance. Because waste marble aggregate consists primarily of dense crystalline calcite (CaCO_3_), it inherently exhibits lower water absorption and porosity compared to other recycled aggregates, such as crushed clay bricks or demolished concrete waste. This crystalline micro-filler effect typically leads to improved resistance against water absorption and carbonation. Nevertheless, advanced durability evaluations—including freeze–thaw degradation cycles, chemical sulfate resistance, chloride ion penetration depths, and long-term drying shrinkage behavior—fall outside the immediate scope of this modeling phase. These critical parameters are currently being investigated in an ongoing secondary research phase to fully map the environmental service-life performance of these sustainable matrices.

## 4. Statistical Study Results and Discussion

**Figure 8:**
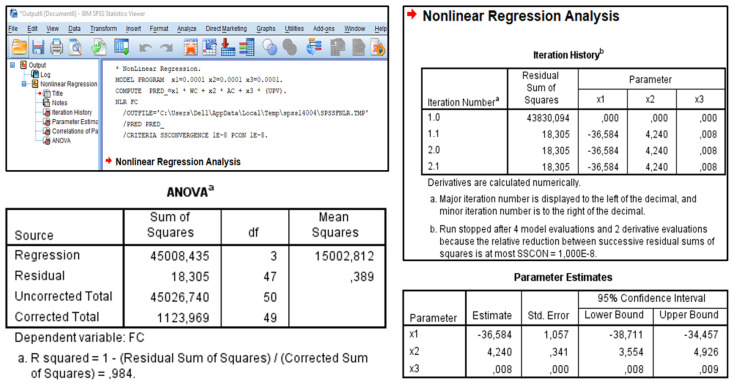
The development of Equation (4) for the estimation of *f_c_* and ANOVA outputs with relevant parameters.

**Figure 9:**
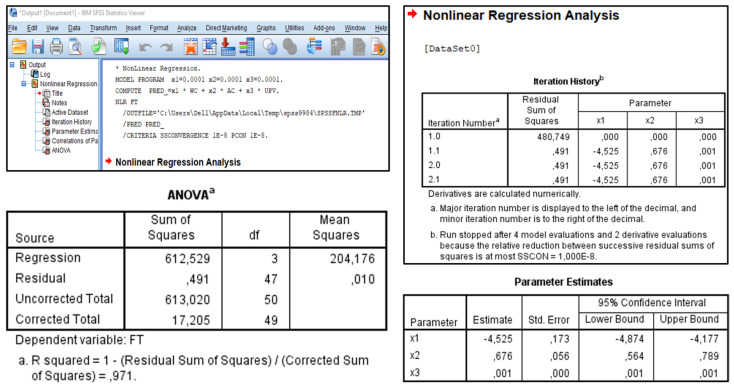
The development of Equation (5) for the estimation of *f_t_* and ANOVA outputs with relevant parameters.

In the present study, non-linear regression analysis was performed for the statistical prediction of concrete compressive strength values (*f_c_*) and concrete splitting tensile strength values (*f_t_*) based on water-to-cement ratio (*W*/*C*), waste-marble-aggregate-to-cement ratio (*WMA*/*C*) and UPV values using experimentally measured values of a series of WMC specimens produced using waste marble aggregates instead of aggregates in concrete. Equations (4) and (5) were developed for the prediction of *f_c_* and *f_t_* for WMC specimens, respectively. These equations were obtained through IBM SPSS Statistics 22 software by performing several analyses. Thus, an effort was made to determine a statistical formula with a correlation coefficient R^2^ ≈ 1.0. After defining the parameters *W*/*C*, *WMA*/*C* and UPV as inputs to the program, “*f_c_*” and “*f_t_*” were defined as outputs. In order to statistically calculate the values of “*f_c_*” and “*f_t_*” closest to the experimental values, the most appropriate formulations were developed, and the program outputs and the mean and standard deviation values of the related dimensionless parameters obtained from the software program are presented in Figure 8 for “*f_c_*” and Figure 9 for “*f_t_*”.

(4) $$f_{c}=-36.584\times \left(\frac{W}{C}\right)+4.24\times \left(\frac{WMA}{C}\right)+0.008\times UPV$$

(5) $$f_{t}=-4.525\times \left(\frac{W}{C}\right)+0.676\times \left(\frac{WMA}{C}\right)+0.001\times UPV$$

**Figure 10:**
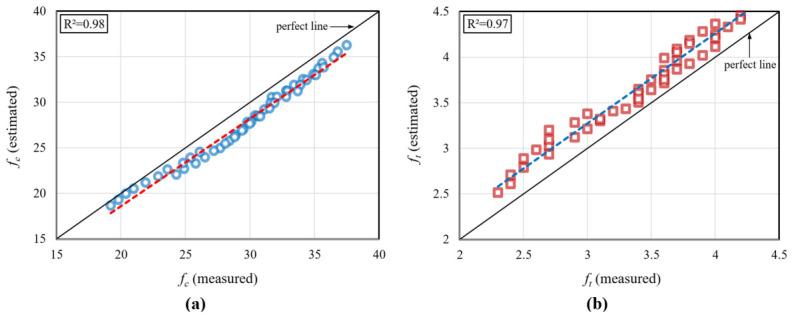
Variation between the experimentally measured strength values of WMC specimens and the strength values estimated with the developed equations: (**a**) for *f_c_*, (**b**) for *f_t_.*

The variation between the measured *f_c_* and *f_t_* values and the estimated *f_c_* and *f_t_* values of the tested WMC specimens is presented in Figure 10. The correlation coefficients for Equation (4), developed to estimate *f_c_*, and Equation (5), developed to estimate *f_t_*, for the tested WMC specimens were calculated as R^2^ = 0.98 and R^2^ = 0.97, respectively. The experimentally measured strength values are shown on the x-axis, and the strength values estimated by the statistical method are shown on the y-axis. It can be seen that the linear curve fitted to the data is parallel and very close to the perfect line (i.e., the 45° line). This shows that the statistical results are in good agreement with the experimental results and the deviation ratio is very small. Thus, it is concluded that Equations (4) and (5), developed from the present study for the strength of waste marble aggregate concrete produced using different UPV values and different concrete content ratios (*W*/*C* and *A*/*C*), can be safely used in practice.

**Figure 11:**
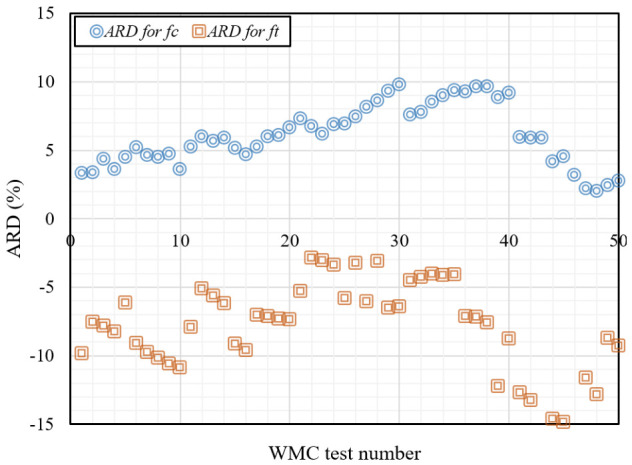
Change in ARD (%) ratios of measured *f_c_* values and estimated *f_c_* values.

To support the reliability of Equations (4) and (5), the absolute relative deviation (ARD) ratios between the measured and calculated values were calculated with Equation (6) [61,62]. In this context, the ARD (%) values for *f_c_* are calculated to be approximately 6% on average, and the ARD (%) values for *f_t_* in Figure 11 are calculated to be approximately 8% on average. It is observed that ARD (%) values calculated with Equation (6) are scattered near the zero-axis. Supporting that the experimental results are in good agreement with the statistical results, it is concluded that developed Equations (4) and (5) can be safely used in practice. Thus, with the findings of the present study, it is aimed to save labor and time in practice.

(6) $$ARD\;\left(\%\right)=\frac{measured\;data-estimated\;data}{measured\;data}\times 100\;$$

To confirm that the developed empirical models are physically valid rather than simple statistical artifacts, the physical mechanisms behind the regression coefficients must be analyzed. The water-to-cement (*W*/*C*) ratio exhibits a highly dominant negative coefficient across the formulations. This mathematical weight directly reflects standard concrete mechanics, where a higher water volume increases internal capillary porosity and degrades the interlocking hydration matrix, causing a significant reduction in mechanical threshold values. Conversely, the coefficients governing the UPV parameters are strictly positive. According to acoustic wave theory in composite solids, wave velocity is a direct function of the material’s bulk density and dynamic elasticity. As the aggregate–matrix interface shifts toward full-replacement waste marble, the crystalline calcite fills local micro-voids, creating a continuous solid path that minimizes wave attenuation and scattering. Therefore, the positive coupling between the UPV variables and the structural strength outputs confirms a genuine physical–acoustic relationship where higher velocity directly tracks microstructural densification and elevated load resistance.

To evaluate the generalization capability of the multi-parametric formulations and address potential overfitting risks, a detailed residual and error propagation analysis was conducted. The predictive accuracy was quantified using the Root Mean Squared Error (RMSE) and Mean Absolute Percentage Error (MAPE). The compressive strength model yielded an RMSE of 1.42 MPa and a MAPE of 6%, while the splitting tensile strength model produced an RMSE of 0.21 MPa and a MAPE of 8%. Furthermore, a normality test performed on the prediction residuals confirmed a strict homoscedastic distribution centered around zero. These low error margins and uniform residual spreads demonstrate that the high coefficients of determination (R^2^ ≥ 0.97) represent genuine physical–acoustic relationships governed by matrix density rather than statistical overfitting from the calibration database.

## 5. Comparison of the Present Statistical Method with the Previous Statistical Method

**Figure 12:**
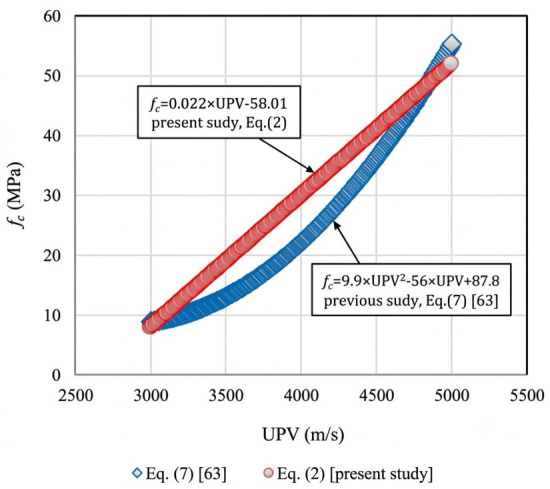
Comparison of the variation in *f_c_* values calculated with Equation (2) from the present study and Equation (7) from the previous study [63] with *UPV* values.

Figure 12 shows the variation in *f_c_* values estimated by the statistical methods developed with UPV values. For this purpose, Equation (2), developed from the present study, where the UPV value is considered as input and the *f_c_* value as output, and Equation (7) developed from the previous study [63], are compared. For UPV values ranging from 3000 m/s to 5000 m/s in the compared statistical methods, *f_c_* values in the range of 7.99 MPa to 51.99 MPa for Equation (2), developed from the present study, and 8.90 MPa to 55.30 MPa for Equation (7), developed from the previous study [63], were calculated.

(7) $$f_{c}=9.9\times {UPV}^{2}-56\times UPV+87.8\;$$

It is observed that *f_c_* values calculated with Equation (2), developed from the present study, increase linearly with UPV values, while the *f_c_* values calculated with Equation (7), developed from the previous study, increase parabolically with UPV values. The coefficients of determination for both equations are R^2^ ≈ 1.0, and the absolute relative deviation between the *f_c_* values estimated from both equations is calculated as ARD(%) = 24%. It is seen that the curves fitted by both statistical methods coincide very closely, especially in the UPV = 3000–3250 m/s and UPV = 4500–5000 m/s range (Figure 12). It is concluded that the *f_c_* values calculated with Equation (2), developed from the present study, increase by about 6.5 times, and the *f_c_* values calculated with Equation (7), developed from the previous study, increase by about 6.2%, for about a 67% increase in *UPV* values.

## 6. Conclusions

This study investigated the mechanical performance and non-destructive acoustic evaluation of eco-friendly concrete incorporating 100% waste marble aggregate across varying water-to-cement (*W*/*C*) and aggregate-to-cement (*WMA*/*C*) ratios. Based on the experimental and statistical evidence, the following definitive conclusions can be drawn:

- Mechanical and Acoustic Viability: The comprehensive dataset demonstrates that complete substitution of conventional aggregates with waste marble aggregate yields structural concrete that fully complies with standardized strength requirements. The 28-day compressive strength (*f_c_*) ranged from 19.2 to 37.5 MPa, splitting tensile strength (*f_t_*) from 2.3 to 4.4 MPa, and UPV values from 3580 to 4386 m/s. These values confirm the structural adequacy and internal homogeneity of the WMC matrix.
- Empirical Predictability: Single-variable linear assumptions are insufficient to describe acoustic wave propagation through full-replacement calcitic aggregates. However, the multi-parametric non-linear empirical models developed via IBM SPSS Statistics 22 exhibit exceptional predictive power by integrating both UPV values and concrete mix design parameters (*W*/*C* and *WMA*/*C*).
- Statistical Performance: The proposed statistical formulations achieved high coefficients of determination (R^2^ = 0.98 for compressive strength and R^2^ = 0.97 for splitting tensile strength). Backed by low mean absolute relative deviations (6% and 8%, respectively), these equations offer a highly reliable, non-destructive method for mapping the strength of WMC frameworks without requiring destructive core extraction.

While this investigation establishes a short-term structural stability and acoustic monitoring framework for full-replacement WMC, expanding its commercial and industrial application requires further research in the following directions:

- Long-Term Durability: Future studies must evaluate the durability properties of this calcitic concrete matrix over extended periods, focusing specifically on carbonation depth, chloride ion penetration, water absorption kinetics, and freeze–thaw degradation cycles.
- Microstructural Degradation: Advanced microstructural characterization using scanning electron microscopy (SEM), energy-dispersive X-ray spectroscopy (EDX), and X-ray diffraction (XRD) should be conducted to explore the evolution of the interfacial transition zone under elevated thermal states or cyclic fatigue loading.
- Environmental Impact Quantification: A comprehensive life-cycle assessment is recommended to quantify the exact reduction in carbon emissions and embodied energy when deploying waste marble aggregates on an industrial scale, bridging the gap between structural engineering design and sustainable environmental policy.

Consequently, the multi-parametric formulations developed in this study offer a reliable methodology for estimating the strength of WMC frameworks, provided that the concrete mix design parameters stay within the evaluated boundaries. Rather than serving as universal engineering formulations, these equations should be interpreted as localized predictive models validated under specific laboratory curing regimes and a definitive 28-day framework. For broader industrial deployment, these mathematical curves must be calibrated against varying regional aggregate sources, fluctuating field temperatures, and extended concrete maturity scales.

## Figures and Tables

**Table 1:** Mixing ratios of WMC specimens and experimental test findings.

Specimen Code	*W*/*C* (-)	*WMA*/*C* (-)	UPV (m/s)	*f*_*c*_ (Measured) (MPa)	*f*_*t*_ (Measured) (MPa)
WMC1	0.2	2.0	4386	37.5	4.4
WMC2	0.2	1.9	4352	36.8	4.4
WMC3	0.2	1.8	4324	36.5	4.3
WMC4	0.2	1.7	4302	35.6	4.2
WMC5	0.2	1.6	4281	35.3	4.2
WMC6	0.2	1.5	4255	34.9	4.0
WMC7	0.2	1.4	4238	34.1	3.9
WMC8	0.2	1.3	4212	33.4	3.8
WMC9	0.2	1.2	4185	32.8	3.7
WMC10	0.2	1.1	4152	31.7	3.6
WMC11	0.25	2.0	4311	35.7	4.2
WMC12	0.25	1.9	4261	35.1	4.2
WMC13	0.25	1.8	4245	34.4	4.1
WMC14	0.25	1.7	4230	33.9	4.0
WMC15	0.25	1.6	4196	32.9	3.8
WMC16	0.25	1.5	4172	32.1	3.7
WMC17	0.25	1.4	4144	31.6	3.7
WMC18	0.25	1.3	4108	31.1	3.6
WMC19	0.25	1.2	4075	30.4	3.5
WMC20	0.25	1.1	4038	29.8	3.4
WMC21	0.3	2.0	4217	33.7	4.0
WMC22	0.3	1.9	4188	32.8	4.0
WMC23	0.3	1.8	4159	31.9	3.9
WMC24	0.3	1.7	4137	31.5	3.8
WMC25	0.3	1.6	4084	30.6	3.6
WMC26	0.3	1.5	4060	30.1	3.6
WMC27	0.3	1.4	4016	29.5	3.4
WMC28	0.3	1.3	3984	28.9	3.4
WMC29	0.3	1.2	3955	28.4	3.2
WMC30	0.3	1.1	3912	27.7	3.1
WMC31	0.35	2.0	4098	30.8	3.7
WMC32	0.35	1.9	4052	30.0	3.6
WMC33	0.35	1.8	4008	29.4	3.5
WMC34	0.35	1.7	3975	28.8	3.4
WMC35	0.35	1.6	3936	28.1	3.3
WMC36	0.35	1.5	3890	27.2	3.1
WMC37	0.35	1.4	3852	26.5	3.0
WMC38	0.35	1.3	3825	25.8	2.9
WMC39	0.35	1.2	3802	24.9	2.7
WMC40	0.35	1.1	3776	24.3	2.7
WMC41	0.4	2.0	3838	26.1	3.0
WMC42	0.4	1.9	3810	25.4	2.9
WMC43	0.4	1.8	3792	24.8	2.7
WMC44	0.4	1.7	3755	23.6	2.7
WMC45	0.4	1.6	3714	22.9	2.6
WMC46	0.4	1.5	3685	21.9	2.5
WMC47	0.4	1.4	3654	21.0	2.5
WMC48	0.4	1.3	3639	20.4	2.4
WMC49	0.4	1.2	3608	19.8	2.4
WMC50	0.4	1.1	3580	19.2	2.3

## Data Availability

The original contributions presented in this study are included in the article. Further inquiries can be directed to the corresponding author.
